# Algorithmic Decision-Making Based on Machine Learning from Big Data: Can Transparency Restore Accountability?

**DOI:** 10.1007/s13347-017-0293-z

**Published:** 2017-11-12

**Authors:** Paul B. de Laat

**Affiliations:** University of Groningen, Groningen, Netherlands

**Keywords:** Accountability, Algorithm, Interpretability, Machine learning, Opacity, Transparency

## Abstract

Decision-making assisted by algorithms developed by machine learning is increasingly determining our lives. Unfortunately, full opacity about the process is the norm. Would transparency contribute to restoring accountability for such systems as is often maintained? Several objections to full transparency are examined: the loss of privacy when datasets become public, the perverse effects of disclosure of the very algorithms themselves (“gaming the system” in particular), the potential loss of companies’ competitive edge, and the limited gains in answerability to be expected since sophisticated algorithms usually are inherently opaque. It is concluded that, at least presently, full transparency for oversight bodies alone is the only feasible option; extending it to the public at large is normally not advisable. Moreover, it is argued that algorithmic decisions preferably should become more understandable; to that effect, the models of machine learning to be employed should either be interpreted ex post or be interpretable by design ex ante.

## Introduction

Two decades ago, Helen Nissenbaum sounded the alert about the erosion of accountability in our computerized society (Nissenbaum 1996). She signaled four trends that obscure lines of accountability. First, computerized systems are usually designed and operated by “many hands.” As a result, responsibility gets diffused over many parties and an analysis of who or what institutions can be held to account is obscured. Secondly, the inevitability of bugs in software, bad enough as it is, also seems to provide an excuse of sorts to stop worrying about faulty software generally. This tends to obscure a proper analysis of bugs in software and hampers the investment of efforts into their elimination and prevention. Thirdly, when things go wrong, all too easily we tend to blame the computer instead of the humans involved. Finally, it is not helpful that software developers flatly refuse to be held to account for their products.

Today, 20 years later, another barrier to such accountability should be signaled. It has to do with the growing importance of decision-making, supported by the use of appropriate algorithms. Algorithms have, of course, been with us for much longer. Two associated trends are new though. Ever more data are becoming available for analysis (big data). This abundance subsequently enables ever more powerful machine learning, an umbrella term for overlapping fields such as pattern recognition, data mining, profiling, knowledge discovery, predictive analysis, and the like (Domingos 2015: chapter one). And machine learning is the tool with which algorithms for a wide array of uses are being created. Let me mention some examples here from both the private and the public sector. Algorithms are the basis for granting loans and setting insurance premiums, as well as for the detection of tax evasion, money laundering, drug trafficking, smuggling, and terrorist activities. Moreover, they steer the answers to our information quests, the advertisements fed to us, the recommendations offered to us, etc.

Such algorithms determine our lives to a great extent. Unfortunately, their outcomes suffer from some notable defects.^1^ Outcomes for the individual may be unjust or differ arbitrarily from one algorithm to the next. On the collective level, outcomes may be biased against specific groups (on the basis of gender, race, and the like); current discriminatory practices may persist in the models used. Most vexing of all, however, is the circumstance that almost all aspects of this algorithmic decision-making remain *opaque*. As a rule, explanation and clarification are hardly supplied. As far as details do come to light, they are usually unilluminating and uninformative. Even legal clauses that address the issue have little effect.^2^ This opacity has become the norm for private institutions. Not surprisingly, they consider their algorithms to be their intellectual property, in need of being kept away from the eyes of their competitors. More surprisingly, public institutions do likewise, shielding their algorithms from the limelight. Joining banks and insurance companies, tax, police, and airport security departments keep the models that they are applying (usually profiles) under close wraps.

It is this opacity that I submit to be the fifth obstacle to proper accountability concerning computerized systems—in particular, those involving algorithmic decision-making. As long as opacity reigns, the discussion about algorithmic decision-making can hardly get started. Notice, though, that even with complete opacity some characteristics of the algorithm involved can be inferred: they can be audited by means of reverse engineering. From the outside, the black box is tested, either by inserting various inputs and observing the outputs, or if input cannot be manipulated, observing the outputs available. Nicholas Diakopoulos presents some case studies of the kind from a journalistic context (Diakopoulos 2015). This auditing, though, is labor-intensive and can only produce limited information about the algorithm.

How to install a culture of accountability? The obvious way forward seems to be breaking the shield of opacity and letting the sunlight in. A call for *transparency* seems justified. Let it be noted that such a call is actually gaining traction these days in many quarters both in the USA and Europe.^3^ With every step towards disclosure, accountability for the functioning of algorithmic systems would seem to come nearer to being restored. Note that I talk here about accountability from an overall moral perspective. An author like Zarsky emphasizes that several other rationales may support a call for transparency. Notably, he mentions the conceptions that government (its civil servants in particular) should be accountable for their actions to be fair and efficient, that informational privacy rights should be respected, and that individuals have a right to know the reasons when decisions adversely affect them (Zarsky 2013: 1533–1550). Useful as this enumeration is, I nevertheless maintain that an overall conception of accountability is the most appropriate here; the rationales he mentions are to be subsumed under this more general umbrella. They are instantiations of the more general conception. Accountability may assume many faces, depending on circumstances; I see no reason, beforehand, to split it up into specified situations of accountability and thereby loose interpretative flexibility.

The analysis of the nexus between transparency and accountability can only proceed after the introduction of two important distinctions. For one thing, several phases of decision-making are to be distinguished (cf. Zarsky 2013: 1523 ff.). First data (sets) are collected (data collection), subsequently the data are used in machine learning in order to develop a model (model development), and finally that model is used for ultimate decision-making (model use). For another, the possible recipients (or beneficiaries) of disclosure are to be distinguished: intermediate bodies that have some oversight role, affected individuals, or the public in general—which obviously includes the former two categories (Zarsky 2013: 1532–1533; cf. also Pasquale 2015: Table 5.1). It is still a matter of discussion what *kind* of institutions could be inserted in between. Zarsky (2013: 1532) suggests that branches of government, or bodies external to it (such as expert panels, NGOs) would do, while Tutt floats the idea of an algorithmic safety agency, a kind of FDA for algorithms (Tutt 2017). Taken together, these two distinctions imply that any call for transparency has to be specific about *what* is to be disclosed and *to whom*; transparency has several gradations.

At first glance, there is a prima facie justification to call for *total transparency*, that is, for the specifics of all phases to be opened up to the general public. Total transparency seems to be the perfect recipe for restoring accountability for algorithmic systems. After all, whenever parties and/or institutions have to be called to account, the “raw data” of the whole process are to be available for inspection. This is the default position from which I start my discussion.

Below, I explore what accounting amounts to when disclosure reigns, for the various phases of algorithmic decision-making. Subsequently, I test my default proposition of total transparency by examining counter-arguments that have been raised against one form of transparency or another. In the literature, four main types of argument are to be found:^4^ (1) Privacy: sensitive data could easily leak into the open; (2) Perverse effects: transparency is an invitation to game the system, making it effectively worthless; (3) Competition: disclosure could hurt companies’ competitive edge; and (4) Inherent opacity: disclosed algorithms are often difficult to interpret, so not much insight is gained. The first three arguments alert us to possible harms resulting from disclosure, while the fourth argument warns us that the benefits of transparency may be limited. From the examination, I ultimately conclude that my default position needs amendment. In particular, at present accountability is not sensibly furthered by making the public at large the beneficiary of full disclosure—only oversight bodies should enjoy full transparency. Moreover, the tension between accuracy and interpretability in machine learning should be subjected to further scrutiny. Preferably, the latter criterion is to be furthered in modeling, even at the cost of the former.

## Ranking Algorithms

Let me first step aside for a moment from the main line of argument and consider the algorithms that we often encounter but have nothing to do with machine learning. I refer here to simple algorithms that intend to rank the performances of hotels, restaurants, airlines, shops, or universities for that matter. Reviews from users involved are solicited for the purpose. Think of TripAdvisor, Booking.com, and Iens or TheFork. Similarly, visitors to social news sites may be asked to vote on contributed content (digging); these votes algorithmically determine the prominence of items on the respective front pages. Think of Digg, Reddit, and Slashdot.^5^

What would accountability amount to? It would mean full transparency concerning all phases of decision-making: the institutions involved are open about how data have been obtained, make these data available to anyone interested, disclose the ranking algorithm in use (down to the very specifics), and specify whether decision-making (i.e., the actual rankings that become public) proceeds in semi-automated or fully automated fashion. But what about the objections mentioned above against such disclosure? Do they apply in these cases? It can easily be shown that they do not.

First, consider the leakage of data deemed to be sensitive. The data here are reviews, ratings, or digs from customers and users. These data, I would argue, should become public par excellence because they are the whole essence around which these systems revolve. So the leakage-objection does not apply.

Secondly, contemplate the possibilities of gaming the system. Could knowledge of the dimensions in use undermine the system as its constituents maneuver to evade them? No, on the contrary: knowing which features count can only induce those involved to do better concerning them. Hotel owners start cleaning their rooms more thoroughly; restaurant owners take care to serve better food; Reddit contributors think more carefully before committing a post, and so on. So, full disclosure of the algorithm has only desirable effects, no adverse ones. Of course another type of gaming is an issue here: constituents may try and manipulate the reviewing system. Multiple (positive) reviews are bought; negative reviews of competitors are fabricated, and so on. So the organizations involved have to put energy into maintaining the integrity of their reviewing system, and build in several checks and controls.

A third objection against transparency was the argument that opacity is needed for competitive reasons. However, as argued above, the ranking algorithms involved just function better when they are out in the open. Moreover, only if these are disclosed, may visitors develop trust in them; would anyone be attracted by secretive ratings? So I would argue that as far as competitive edge is concerned, it is fostered rather than eroded by transparency.

Finally, we have the non-interpretability objection. It does not apply here; without exception, the models in use here are easy to understand, essentially just a (weighted) summation of ratings along several dimensions.

In practice, the organizations employing such rankings do indeed open up details of their workings, to varying degrees.^6^ Of course, this transparency also opens up avenues for discussion and dissent. What criteria do users have to fulfill before being allowed to commit a review? Why use this dimension in the algorithm and not another? Does the algorithm work as intended? But that is how it should be: a spirited and open public discussion.^7^

## Predictive Algorithms

This was a preliminary analysis that I presented by way of contrast. As soon as predictive algorithms produced by machine learning are involved, the issues are more complicated. This mainly has to do with the character of the algorithms involved. The variables involved are no longer constitutive of rankings, they are indicators supposed to *predict* target variables*.* User reviews of restaurant food score the quality of the food itself; histories of credit card use merely serve to predict whether someone will repay a loan in the future.

This difference has huge consequences for accountability, for all phases of decision-making it becomes much more intricate. What would it amount to if my default position of introducing total transparency applies? What would the shape of accounting become?

Before going into the issue, a preliminary remark is due. Machine learning is very much interrelated all through the stages of data collection, model construction, and model use. When calling an organization to account concerning, say, the use of a profile, an account of what the profile means and how it has been constructed is inescapable. And any account of how an algorithm has been constructed, cannot do without an account of how datasets have been used in the process (say, as concerns possibly biased data). So accounting for machine learning models can only make sense if all phases are taken into account.^8^ With this conception, I take aim at Zarsky who painstakingly tries to relate the rationales for transparency to disclosure of specific stages of decision-making (Zarsky 2013: 1533 ff.). I think that in the domain of machine learning in which all phases are tightly interrelated, this is a futile exercise. Transparency only makes sense if all aspects of machine learning are laid bare.^9^

Now let me return to the task at hand and analyze the meaning of accountability as soon as full transparency is observed.^10^

### Phase 1: Data Collection

At first, datasets have to be collected which are to serve as input to the process of machine learning proper. The quality of them is absolutely essential since any shortcomings risk being built into the very model to be developed later. An obvious requirement is that data are appropriate to the questions being asked. Frictions of the kind may arise in particular when data from one context are imported into another context—the data need to be reinterpreted which is a precarious task. As a case in point compare the sale of prescription data from pharmacies to insurance companies; these were to be used in a machine learning effort to predict health risks (Pasquale 2015: 27). More generally, this addresses the importance of careful scrutiny of the practices of data brokers, companies that buy as many datasets as possible from everywhere, and resell them to any interested party.

A particular concern that of late has attracted a lot of attention is, whether the datasets are free of bias.^11^ For the sake of illustration, let us consider model construction for admission decisions concerning university education. Apart from the issue that the target variable (fit for education) is a subjective affair, the process of labeling applicants with one of its possible values (“class variables”—in this particular case either “fit” or “unfit” for education) may be influenced by prejudice, say against women. So from the very start, some of the training data points may carry wrong labels (Barocas and Selbst 2016: 681 ff.). Furthermore, the dataset to be used for training may be biased against specific groups that society wants to protect from discrimination. Along, say, lines of race or gender, records contain more errors, less details, or simply suffer from underrepresentation in the sample as a whole. Unavoidably, skewed data will produce a skewed model later on (Barocas and Selbst 2016: 684 ff.).

Moreover, only very coarse features may be used in model construction (for the sake of cost efficiency), while these features demonstrably correlate with sensitive dimensions like race or gender. Compare the infamous practice of “redlining”—simply excluding neighborhoods as a whole—in deciding about granting credit. Ultimate decision-making will reproduce this bias (Barocas and Selbst 2016: 688 ff.). Finally, the dataset may contain variables that serve well for predictive purposes, but at the same time correlate with one or more sensitive categories. This so-called proxy-problem is a hard one: how to distinguish between the discriminatory and the non-discriminatory part (Barocas and Selbst 2016: 691 ff.)?

For all of these defects in training data, one has to find remedies to be applied in the subsequent phase of model construction (see below: “discrimination-aware” modeling).

### Phase 2: Model Construction

Subsequently, the data available are used as training material for machine learning. The techniques employed are various: classification and decision trees, support vector machines (SVMs), ensemble methods, neural networks, and the like. In inductive fashion, an appropriate model gets constructed that best fits the data. Such a model evolves step by step, its error ever diminishing.^12^ Models are made for purposes of prediction: think of predicting who deserves a loan, what insurance premium to set, whom to inspect for tax evasion or for suspicious activities at the airport, and so on (cf. above).

By way of illustration, take the construction of a decision tree. In recursive fashion, the training data are split ever and again into subsets (nodes) along a single attribute. At every step, one chooses the attribute that best separates the data at hand. What criterion to employ for splitting? A common measure for determining the best separation is a variable called “information gain”: the difference between the amount of “entropy” before and after the contemplated split (summated with proper weights). The highest information gain indicates where the next split should take place. While proceeding in this fashion, the amount of entropy decreases with every step. The procedure stops when all subsets are pure (all elements belonging to a single class)—and hence entropy has become zero for all of them.

In the process of modeling, several pitfalls have to be avoided. A dominant concern is “overfitting”: one goes on and on to train (say) the classifier until the very end. The end product surely fits the training data—but only those; it is unfit to generalize to other, new data.^13^ One recipe against overfitting (among many) is to divide the training data into a training set (80%) and a test set (20%). The classifier is trained on the first set, its error diminishing with every iteration. Simultaneously, one keeps an eye on the classifier’s error as applied to the test set. When the latter error starts to increase, it is time to stop and be satisfied with the classifier a few steps back (early stopping). In another approach, one fully grows the classifier, but subsequently, working bottom-up, prunes it back until the so-called generalization error (on a test set) no longer improves.

More generally, one may try out several classifiers simultaneously. For the purpose, divide the available data into training set, validation set, and test set. Then train the classifiers on the training set, choose between them by comparing performances on the validation set, and characterize the performance of the chosen classifier by applying it to the test set.

Note that most procedures in machine learning as just described are what its practitioners call “greedy”: they select the local optimum at each step of construction; hence, global optimality is not guaranteed. Therefore, machine learning is not so much a science in search of the unique solution. It more resembles the art of engineering which tries to find a workable solution; good judgment and intuition are needed to steer toward a good-enough model.

A further problem that needs to be taken into account is the “class imbalance problem.” In many areas, the class variables of the target variable are represented very unequally in the population. Think of transactions that amount to tax evasion, monetary fraud, or terrorist intentions—these only make up a tiny fraction of all transactions. Training on such an imbalanced dataset may produce a model that over fits to the majority of data representing bona-fide transactions. In order to circumvent the problem, a conditio sine qua non is choosing an appropriate performance measure, ensuring that false negatives are given more weight than false positives.^14^ Besides, the main approach is to adjust the available training set in order to obtain a more balanced set. Either one deletes data points from the overrepresented class (undersampling) or adds data points from the underrepresented class (oversampling)—for a recent overview, cf. Chawla (2010). The latter alternative, of oversampling, can also be implemented in a more sophisticated fashion by artificially creating new data points that are located nearby the available minority data points (SMOTE, as originally proposed by Nitesh Chawla in Chawla et al. 2002).

A final accounting task that needs mentioning here relates back to my discussion of bias in underlying datasets. If such data are used inadvertently for model construction, chances are that the biases involved will be built straight into it. Concerns of the kind have generated efforts towards “discrimination-free” or “discrimination-aware” modeling.^15^ At first sight, it would seem that simply deleting any sensitive dimension involved from datasets would be a step forward. However, some of the remaining model variables may actually be correlated with it, allowing discrimination to continue. In consistent fashion, one may take the next step and eliminate all correlated dimensions as well. But at a price: every deletion of a variable also deletes information valuable for the task of prediction.

In order to prevent this loss of information, these practitioners prefer to keep biased datasets *intact*. Instead, the very models and their datasets for training are being reconsidered. How to train models with a view to obtaining unbiased results? In the pre-processing stage, one may change the set of training data involved. Options to be considered are locally “massaging” the data in such a way that borderline cases are relabeled, and/or locally introducing “preferential sampling” that deletes and/or duplicates training instances. In the processing stage, one may take to developing models under non-discrimination constraints. In the post-processing phase, finally, one may try and suitably alter the classification rules obtained. Such deliberations about circumvention of bias in modeling should become part and parcel of the accounting process concerning model construction.

Notice that accounting on this score may benefit from developments in the fresh field of “algorithmic transparency.” These include procedures to test models of machine learning, afterwards, whether they suffer from “algorithmic discrimination” or not. Testing consists of randomly changing the attributes of the sensitive dimension in the training set (say, from male to female and vice versa). “Quantitative Input Influence” measures allow an estimation of whether or not group properties (like race or gender) have undue influence on outcomes (Datta et al. 2016).

### Phase 3: Model Use

Upon completion, the model is ready to be used for making decisions. That is, for decisions about granting that loan or charging that insurance premium, or about whom to inspect for tax evasion or for suspicious activity, and the like. Such decisions can now be made *assisted* by the algorithm developed; by no means it is implied that these are to be taken in fully automated fashion. As a rule, there is an array of possibilities: from mainly human to fully automated decision-making. Depending on the particular context at hand, one or other solution may be optimal. In the context of camera surveillance (CCTV), for example, Macnish devotes a whole article to arguing the various options; in conclusion, he recommends a combination of manual and automated modes of making decisions (Macnish 2012). So I want to argue that at the end of the day, proper accounting should provide a reasoned report about and justification for the chosen levels of automation in decision-making.

## Objections

With this sketch of accountability for algorithmic decision-making under conditions of full transparency, my default position has been clarified. It is now time to go into the objections raised to transparency and examine whether or not they yield valid arguments for toning down transparency. In particular, reducing the transparency of some constitutive elements of the algorithmic process (say, keeping the algorithm opaque) or restricting the beneficiaries of transparency (say, excluding the general public).

Above, I mentioned four kinds of objections. The first one was the privacy objection that data could easily leak into the open. This may conveniently be tackled first. If full transparency also pertains to the very datasets involved in machine learning, one can easily imagine scenarios in which these, after having been queried by interested individuals or groups, are simply diverted from the intended purposes and leak away to be used for other purposes. We already live in a big data society where datasets are routinely distributed or sold to third parties. Data brokers make a living from the practice. There is no need to furnish more fuel for those practices. So it would seem that this privacy argument calls for some restraint. In particular, prudence suggests that the datasets involved are only to be made available upon request to intermediate parties that have oversight authority of a kind. The public at large is not to be entrusted with the power to scrutinize datasets as used in machine learning (although the entries of individuals should of course be open for personal inspection). But the restraint stops right there; oversight boards should have full authority to summon up whole databases, whether from public or private parties, for purposes of, say, auditing the machine learning involved.^16^

With this restriction in mind, the other three objections to transparency—possible perverse effects, loss of competitive edge, inherent opacity—can now be addressed. As will be shown, these are much tougher to deal with.

## Perverse Effects

A second objection raised to total transparency warns us against various perverse effects. Most prominent is the argument about “gaming the system”: as soon as a model is in the open, interested parties may be able to detect the proxies involved and subsequently evade them. If that happens, the value of the model employed obviously diminishes considerably. Various telling examples can be found dispersed in the literature (or, for that matter, discovered by one’s power of imagination). Potential tax evaders search the red flags that figure in profiles of tax evasion (e.g., membership of certain occupations, high donations to charity). Consumers in search of a loan find out that the use of credit cards is held against them. Potential terrorists learn that paying for a plane ticket in cash and carrying a knife are both considered useful proxies for evil intentions, and that being a Muslim of Middle Eastern appearance is even more of a red flag for terrorist inclinations. As can be seen, proxies may relate to any kind of behavior (whether lawful or not), as well as to personal characteristics (cf. Zarsky 2013: 1557 ff.). Machine learning is agnostic in this respect and will digest any information fed into the machine.

Gaming, then, refers to behavior that intends to evade the proxies concerned: omitting the mention of one’s occupation, suspending charity donations, getting rid of one’s credit cards, paying for the airplane by other means, leaving knives at home, or asking a non-Muslim colleague from elsewhere to take over. From this list, it transpires that any evasion carries costs, from small to large, and the potential evader must weigh them against the potential benefits from the evasion. My last example—of the potential Muslim terrorist—also teaches us that some proxies cannot be evaded so easily (personal characteristics).^17^

Whatever the context, gaming is to be reckoned with. It potentially undermines the algorithm’s accuracy. Are there ways of having an algorithm in the open, without gaming leading to its demise? One solution, obviously, is to evade the issue and rely only on those proxies that are immune to manipulation. Another solution accepts the challenge and aims to dynamically take manipulation into account while modeling. A new research effort of the kind has started from the intuition that gaming carries a cost; so, fighting the “gamers” should be possible via manipulation of the costs they incur. It is this intuition that drives an emerging field that combines classification with game theory. It tries to develop dynamically changing classifiers that respond to the moves of their adversaries. The “adversarial prediction games” involved are helpful in making models more robust against manipulation of proxies. First results (concerning spam filtering) indicate that such dynamic classifiers perform somewhat better against gaming adversaries than static ones (Brückner et al. 2012; Hardt et al. 2016).

While this direction is mainly a promise for the future, another approach to curb gaming the system can be implemented any time: preventing the disclosure of said proxies to potential gamers. The public at large is no longer to be a beneficiary of transparency concerning the algorithm in use. Of course, individuals subjected to a decision from one algorithm or another should keep the option to receive a transparency report about that particular decision. That might give him/her some clues for future gaming, but only to a very limited extent. Obviously, intermediate oversight authorities are to retain full rights of transparency as far as the model and its proxies are concerned, otherwise all accountability is gone.

Zarsky warns against another perverse effect of transparent models (Zarsky 2013: 1560 ff.). It concerns the use of personal characteristics one cannot escape from. So far as they touch upon societal sensitivities (race, religion, ethnicity, nationality, and gender), their use in modeling carries the danger of *stigmatization*. By way of summary illustration, let me take the case of black people in the USA (or elsewhere). For one thing, harassment by, say, traffic controls based on race as a proxy may intensify existing feelings of being discriminated against. For another, incorporating the black feature as a proxy in modeling signals to non-blacks that something is amiss more generally with their black fellow citizens. As a result, the social divide only widens from both sides.

To prevent such unwanted escalation, several options present themselves, which are actually quite similar to the ones to evade gaming. For one thing, modelers may try to evade the issue and delete all sensitivities from the model, but at the risk of deleting valuable information. For another, modelers may take to discrimination-aware modeling that tries to compensate ex ante for discriminatory effects as reflected in the data to be used for machine learning. As may be recalled, such advice had already been incorporated in best practice rules for practitioners of machine learning (cf. above). With this option, it seems imperative to clearly communicate to the public at large that anti-discrimination measures have effectively been taken—which, on the face of it, seems a difficult task in view of the sophistication of the modeling involved. A third option, obviously, always remains available: prevent the issue of stigmatization from arising at all and restrict transparency concerning the algorithms in use to oversight bodies alone, so far as they exist.

## Competitive Edge

A third type of argument against total transparency of the algorithmic process is an argument about property: companies that develop and maintain algorithms may consider them to be their intellectual property. Ownership supposedly guarantees them the edge over competitors who operate with similar algorithms. Ways to implement this property entitlement are keeping them a secret (trade secrecy) or applying for a patent. Although such patenting is on the increase (especially by Google), secrecy is usually preferred since the field of machine learning is moving rapidly.

At first sight, this argument seems to pertain to the private sector only; for public uses, it seems to be irrelevant. However, one has to take into account that many public services (such as collecting taxes, border control, and airport security) employ software bought on the market, or outsource the whole process to companies. So the reach of this property argument is vast.

What to think of this argument? One could argue against it that open source innovation (for software in particular) has proved to be viable for companies. Firms may well survive and prosper in a completely open environment; they just have to be better and faster than their competitors. This should also be possible for machine learning algorithms in transparent settings. It is abundantly clear, however, that not many companies buy this stance on innovation; competition and innovation on closed terms have remained the norm. As a result, only complicated and protracted political and regulatory battles may achieve changes on this score.

In the light of these diverging visions, only limited progress seems feasible. A possible scenario is the following. As far as public interests (broadly construed) are concerned, society no longer accepts that algorithms remain completely secret, and therefore unaccounted for. Balancing this requirement with the proprietary instinct of companies, an acceptable compromise is to limit full transparency to intermediate parties only. Oversight boards, tied to secrecy, may inspect and control the various algorithms the public is subjected to and dependent on. Accountability takes place behind closed doors. After this first step, private companies may possibly be pressed at a later stage to conform to the same regime of transparency and open up their algorithms to oversight boards as well.

## Inherent Opacity

A final objection against transparency, especially of the very algorithms themselves, is to point out that algorithmic end-products of machine learning are often difficult to interpret, even by experts. The algorithms hopefully yield accurate outcomes, but an explanation in understandable terms as to why a specific decision is recommended cannot be supplied. The model is effectively a black box for all of us, laymen and experts alike. This is the problem of “interpretability” or “explainability” as it is called. Hence, the argument continues, such transparency delivers very little in terms of explanation. It can only yield technical clarifications about the classificatory accuracy of an algorithm, but it cannot clarify the reasons behind its particular recommendations.

What about the truth of this assertion? Classifiers, or decision trees, have always been easily interpretable. By their very construction, any observer can go along the tree from top to bottom and have an inkling of how input variables influence the output. Gradually, however, the techniques involved have become ever more sophisticated. In the search for more accurate predictions “ensemble methods” have been developed; interpretability has suffered as a result. Let me present a few of these methods.

*Boosting*, as invented by Robert Shapire in the 1990s, is a technique for optimizing a particular classifier (say, a decision tree) (Freund and Shapire 1999). One repeatedly calls the same algorithm on the training data, but these are altered ever so slightly. After each round, one inspects how well data points have been classified; those that were classified wrongly are given greater weights in the next round. After typically 25 to 50 rounds, one stops. The final classifier is obtained by the summation of all classifiers that have been produced, weighted by how well they actually performed in classifying the (weighted) data points. Such a summation effectively obfuscates interpretation.

Another technique for optimizing a classifier is *bagging* (short for bootstrap aggregating), as invented by Leo Breiman in 1994 (Breiman 1996). The algorithm is called repeatedly, typically several hundreds of times, and each time applied to a fresh subsample (of fixed size) of the training set as a whole—with subsamples being drawn *with* replacement. In the end, all classifiers obtained vote on the outcome for each data point; so effectively, the majority vote of all classifiers together decides. No weights have to be calculated as in boosting. What happens, intuitively, is that random variations in the dataset cancel each other out, leading to more accurate classification. As with boosting, interpretability suffers.^18^

In a subsequent development, bagging of classifiers has been modified by Tin Kam Ho. While repeatedly training the algorithm, introduce what has been named *feature bagging*: at each round do not involve all features of the training data, but select a fresh random subset of features from them (its size being a fixed number) (Ho 1998). As in the case of “normal” bagging, the majority vote decides the outcome. This “random subspace method” aims to contain the dominant influence of strong predictors.^19^ While this is again a summation method, interpretability is sacrificed once more.

So, in a nutshell, modern classifiers no longer employ just one single tree for classifying fresh data, which would allow easy interpretation. Instead, they use multiple trees, up to hundreds of them, in a summation procedure. Then, it is no longer feasible to pick any tree from the forest as being the most important one. As a result, the explanation of classification outcomes (which factors contributed most to a particular result?) is no longer a straightforward affair.

Next, consider neural networks. These have always been inscrutable by their very design. In them, a middle layer (or more than one of them) is inserted between input and output. The weights connecting input variables to the middle variables as well as those connecting the middle variables to the output variable are being adjusted in several iterations. The end model obtained displays all those weights, but cannot be interpreted as to how much the various input variables contribute to the outcome. A similar remark applies to SVMs. This method focuses on the construction of a hyperplane separating the classes of the target variable (say, the + 1's from the − 1's). Whenever a linear solution is unfeasible, one applies the “kernel trick” that projects the training data into a higher dimensional space. Subsequently, in that space, a solution plane can be found in linear fashion, but interpretation of the parameters is a hard task.

Finally, it should be signaled that increasingly classifiers—as well as neural networks—in use are being updated dynamically. As new data pour in, the opportunity presents itself to update the classifiers or neural networks involved. With the increase in big data and computer power, this trend is inescapable. As a result, if interpretation is feasible at all, it is bound to change all the time.

In sum, the search for increasing accuracy pushes interpretability into the background. What can be done to overcome this trend? Can interpretability be salvaged? Two directions, rather outside the mainstream of developments, are being suggested by practitioners.

The first option is to try and recover interpretability *after* the fact—i.e., once classifiers have been obtained, one tries to test them and find elements of interpretation, in spite of their prima facie appearance as a black box. I am aware of two initiatives in this vein. For one thing, practitioners advocating “algorithmic transparency” have developed “Quantitative Input Influence” measures; these are intended to show how much individual variables (as well as combinations of them) have contributed to the final algorithmic outcome—say of being rejected for a loan (Datta et al. 2016). These transparency indicators are supposed to be applicable to a wide range of classifiers and SVMs.

For another, David Barbella and co-workers try to develop ways of interpreting SVMs ex post (Barbella et al. 2009). For any particular data point, they strive to find the nearest “support vectors.” These are border points near the hyperplane that give an indication of how close the particular individual has been to being classified otherwise; say, to obtaining the loan if the applicant’s request has been rejected. Alternatively, a list of support vectors can be generated, ordered by the strength of their pull on the individual involved. Border classification, finally, gives an indication of how much parameters would have to change for a particular test point to be located at the hyperplane, and thus, end up in the other category (say, again, of obtaining that loan).^20^

The other more fundamental option to salvage interpretability is restricting one’s machine learning efforts beforehand and only working with those methods that are, from their very design, easily interpretable. Of course that limits one’s options considerably, given that the main trend is in the opposite direction. Most of the time, this implies that new models have to be built. In that vein, scientists such as Cynthia Rudin and co-workers operate, advocating the use of models that produce so-called Bayesian Rule Lists—which are actually one-sided decision trees (Letham et al. 2015). The preferred length of the rule list and the preferred number of conditions per rule are specified beforehand (in accordance with the wishes of the particular user). The goal of the method is to show which variables causally contribute to a particular prediction, and how much (in the case of Letham et al. 2015: to the occurrence of a stroke). A series of if/then statements guides us through the model. The authors argue that, in the medical context in particular, black box decisions about health issues are no longer acceptable to medical personnel and patients alike.^21^

Summarizing, in machine learning, one either goes with the flow of increasing accuracy and thereby sacrifices explanatory power for the subjects involved; or one goes against it and stresses interpretation ex post of current models and/or restriction ex ante to easily interpretable models. The first option (of increasingly accurate models) effectively reduces accountability to a merely technical exercise, incomprehensible even to experts (though important enough for answerability), while the second option (of foregrounding interpretability) opens up possibilities for fully fledged accountability. Therefore, to anyone who cares for answerability, efforts to salvage interpretability are worth pursuing. This statement roughly resonates with the leading opinion in legal circles both European and American concerning the importance of adequate explanations. The most recent EU Data Protection Regulation (General Data Protection Regulation, GDPR, Regulation (EU) 2016/679) grants its data subjects the right “to obtain an explanation of the decision reached” when automated processing is involved (preamble 71), while the Fair and Accurate Credit Transactions Act of 2003 (FACTA) requires American credit bureaus to provide a consumer upon request with a list of “all of the key factors that adversely affected the credit score of the consumer in the model used” (Sec. 212).^22^

Suppose we go for the second option. In that case, the objection that the inherent opacity of machine learning renders the disclosure of algorithms an empty gesture that yields no more than a technical check on their accuracy, vanishes into thin air. Transparency of machine learning algorithms can contribute to making the practitioners involved fully answerable to society for their practices—provided that their motto is “interpretability first.”

## Conclusions

The default position I started from was full transparency of the whole algorithmic cycle all through to the public at large. Consideration of the various counter-arguments uncovered reasons to override this position. First, for the sake of privacy it would be unwise to make underlying datasets freely available to anyone; it would amount to an invitation for violations of privacy. Secondly, full transparency concerning the machine learning models in use may invite those concerned to game the system and thereby undermine its efficiency. As long as indicators in use remain non-robust against manipulation, the only remedy as yet is, again, excluding the public at large from obtaining full transparency. The same conclusion applies to models that may provoke stigmatization; restricting their disclosure to oversight bodies alone seems indicated. Thirdly, as a rule, companies emphatically emphasize their property rights on algorithms. As long as this stance continues to be held, the only feasible alternative to complete opacity is, again, transparency limited to intermediate parties involved in oversight (concerning algorithmic decision-making for public purposes, to begin with).

The three counter-arguments can be seen to work in unison towards similar kinds of restrictions. Bombarding the public at large to be the beneficiary of full transparency would, so to say, perversely affect accountability. We should normally satisfy ourselves with oversight bodies enjoying full transparency; they are to perform the task of calling algorithmic systems to account in our stead. In conjunction, affected individuals should be entitled to obtain a full explanation about decisions that concern them. Oversight bodies may help to ensure that those accounts are indeed procured.

Meanwhile, the important issue of interpretability of algorithms continues to loom large. Do we remain on the dominant trajectory of developing algorithms that become ever more accurate but ever less intelligible? Or do we opt instead for another trajectory of requiring explanations for decisions, which can be accomplished either by making model outcomes understandable ex post, or by choosing models ex ante that are intelligible by design? Only the latter trajectory would enable full accountability, to experts and laymen alike.^23^

These are sobering conclusions about the limited amount of transparency in algorithmic decision-making that is feasible nowadays, given the state of the art in machine learning and the current political climate. Nevertheless, in the above, I indicated several tendencies that may challenge these conclusions. Whenever, in the future, algorithms in use routinely will be designed as robust against manipulation and intelligible by design, room will be available for enlarging transparency further. Then it would make sense for algorithms (and their outcomes) to be opened up for perusal to the public at large; accountability for them no longer needs to be implemented behind closed doors only. For this development to materialize, however, political pressure will have to be exerted on companies to let them relinquish their property claims on the various algorithms at issue—a tall order no doubt.

I commenced this article by mentioning Helen Nissenbaum who, two decades ago, signaled four barriers to accountability for computerized systems. Dealing with the opacity of algorithmic decision-making as the fifth obstacle of the kind has turned out to be a thorny issue that needs a lot more theoretical and political effort before progress can be made.

## Footnotes

1 For credit scoring, for example, cf. Citron and Pasquale (2014).

2 Cf. Zarsky (2013): 1510 ff., 1523 ff. for the situation in the USA.

3 Specifically, the call for “algorithmic transparency” is being discussed in circles of academics, professionals (ACM), governments (FTC, the White House), parliaments (EU), and activists (EPIC).

4 See Zarsky (2013): 1553 ff.; and Kroll et al. (2017): 23–24. Many other authors mention—some or all of—the counter-arguments in passing.

5 Site ranking by a Google search is not included here. Though it started as simple ranking (by means of the PageRank algorithm, now patented), ranking is now governed by the more complex “personalized search” which embeds one’s search terms in one’s own personal context (Outride system; cf. Pitkow et al. 2002). Moreover, the search engine is continuously involved in a guerrilla war against manipulators of rankings. Also, sites may appear at the top of the list of results by “paid inclusion.”

6 For example, the restaurant ranking sites Iens and TheFork (all acquired by TripAdvisor) use as criteria for rating: food quality (50%), service (25%), and ambience (25%). The score of a restaurant is calculated as the average of scores by customers over the last year. Moreover, all reviews—ratings and wording included—are made available for public scrutiny.

7 Compare the heated discussion in Reddit whether the algorithm for the front page functions as intended: does it by any chance suppress fresh posts immediately after they are voted down by just a few reviewers?! See https://bibwild.wordpress.com/2012/05/08/reddit-story-ranking-algorithm/, and http://www.tuicool.com/articles/JjQ7Rr.

8 By way of example: if my request for a loan is denied, I could ask for the reasons for the decision. If these turn out to relate to my history of credit card use, I may go on and ask how that variable figures in the developed profile as a whole. Upon discovering that the factor race is part of the profile, I may continue and inquire whether discrimination-free modeling has been applied to the training data.

9 With a possible exception for the underlying training data themselves–see below.

10 Notice that in the sequel I assume that the issues of consent and privacy concerning datasets have adequately been taken care of—which is obviously a large assumption. Moreover, I sidestep the thorny issue of distributing responsibility over the many hands (algorithms included) involved in the process; my subsequent discussion of various objections against transparency can proceed without having sorted out this issue.

11 In the sequel, I extract the most salient points from Barocas and Selbst (2016).

12 With tasks of classification, this error is usually characterized by the measures of precision and recall (often combined into the F-measure).

13 Another vexing problem is the “curse of dimensionality”: our intuitions and our methods soon fail with an increasing number of dimensions (cf. Domingos 2012).

14 Usually, the already mentioned measures of precision, recall, and the F-measure will do in this respect (cf. above, note 12).

15 Cf. the edited volume about this and related issues: Custers et al. (2013); in particular, the volume chapters Kamiran and Zliobaite (2013); Kamiran et al. (2013); and Hajian and Domingo-Ferrer (2013). A useful summary concerning prevention of discrimination in modeling is to be found in Romei and Ruggieri (2013): 622–624.

16 By way of example, when an auditor wants to perform a check on algorithmic discrimination, these very data are needed to perform the tests (cf. above).

17 Zarsky (2013: 1558-1560) also mentions a similar threat—as originally signaled by Harcourt: knowledge of proxies may encourage those who fall *outside* their range, to commit the very infraction the model is trying to detect. Say the taxpayer who is not a member of any of the red-flagged occupations decides it is safe to try and evade taxes. I omit it from my discussion since it is a rather far-fetched phenomenon; at any rate, the remedies are the same as against gaming.

18 Breiman himself already expressed worries of the kind: “What one loses, with the trees, is a simple and interpretable structure. What one gains is increased accuracy” (Breiman 1996: 37).

19 The combination of “normal” bagging and feature bagging yields the so-called *random (decision) forest* method.

20 The authors also mention similar initiatives such as sensitivity analysis, inverse classification, and rule extraction (Barbella et al. 2009: 1–2).

21 In this respect, Datta et al. (2016) also recommend super sparse linear integer models and probabilistic scaling.

22 Cf. Citron and Pasquale (2014): 17–18. Notice though, that the various clauses hardly seem to be adhered to in practice (cf. Schermer 2011: 51).

23 My conclusions turn out to be comparable to those reached by Zarsky (2013: 1563 ff.) who, let it be recalled, investigated governmental decision-making—though his at times tortuous phrasing makes the comparison a hard task. The difference between us is one of emphasis. While Zarsky treats the various phases of algorithmic decision-making in greater detail, I delved more deeply into the possibilities of developing algorithms that are robust against gaming, and the tension in machine learning models between accuracy and interpretability.
